# Improvement of culture and acclimation conditions in a bio-nursery system for *Paeonia lactiflora*

**DOI:** 10.5511/plantbiotechnology.25.0120a

**Published:** 2025-06-25

**Authors:** Kazuhiko Yamamoto, Takayuki Inui, Noriaki Kawano, Takayuki Tamura, Miki Sakurai, Tomokazu Jinbou, Katsuko Komatsu, Kayo Yoshimatsu

**Affiliations:** 1 Tsukuba Division, Research Center for Medicinal Plant Resources, National Institutes of Biomedical Innovation, Health and Nutrition; 2 Center for Medicinal Plant Resources, Toyama Prefectural Institute for Pharmaceutical Research; 3 Botanical Raw Materials Cultivation Technology Development Department, Botanical Raw Materials Division, TSUMURA & CO.; 4 Institute of Natural Medicine, University of Toyama

**Keywords:** bio-nursery system, medicinal plant, *Paeonia lactiflora*, plant tissue culture, seedling propagation

## Abstract

*Paeonia lactiflora*, the roots of which are used as a crude drug, is one of the most widely used and important medicinal plants. The long cultivation period and low proliferation rate of *P. lactiflora* makes it difficult to propagate large numbers of plants within a short period. We developed a bio-nursery system using plant tissue culture techniques to contribute to the supply of *P. lactiflora* seeds and seedlings in Japan. Here, we report on the improved tissue culture and acclimation conditions for a more stable and efficient bio-nursery system. We investigated the effect of culture conditions on shoot proliferation and the effect of calcium concentration during root induction and acclimation of cultured plantlets. The results demonstrated that the number of shoots increased under the 15/5°C diurnal temperature changing treatment [15°C, 12 h light (fluorescent light, 80–130 µmol m^−2^ s^−1^)/5°C, 12 h dark] compared to a constant temperature of 15°C. A higher calcium concentration (6 mM Ca^2+^) during root induction resulted in more vigorous growth after transplantation to the soil. In addition, it was found that planting in a closed greenhouse at a constant temperature of 20°C after cold treatment was suitable for acclimation of cultured plantlets. These findings are expected to contribute to the future seedling supply of *P. lactiflora.*

The peony, *Paeonia lactiflora* Pallas, is a perennial plant in the family Paeoniaceae, and its dried roots are used as a crude drug (The Ministry of Health, Labour and Welfare 2021), and are included in many Kampo formulas. The peony is in high demand as a cut flower because of its beautiful and fragrant flowers. Furthermore, peony flower extracts have recently been reported to have antioxidant and anti-tyrosinase activities and are used as cosmetic ingredients (Zhou et al. 2023). Peony roots, which contain several compounds such as paeoniflorin and albiflorin, are used for its astringent, antispasmodic, and sedative properties (Namba 1993). The demand for peony roots in Japan is high, and the amount used in the fiscal year 2020 was approximately 1,712 t (Yamamoto et al. 2023), the third-highest quantity after glycyrrhiza and poria sclerotium. Although the use of peony roots in Japan is increasing each year, approximately 98% of its supply is imported from China. Hence, there is a strong demand to expand the domestic cultivation of peonies in Japan.

The cultivation of peonies used in herbal medicine requires more than three years when grown from rhizomes and more than six years when grown from seeds. Although peonies are usually propagated by dividing its rhizomes, it is difficult to propagate large amounts in a short period because of the long cultivation period and low propagation rate. Therefore, establishing a sustainable supply system for peony seeds and seedlings is a major issue.

There have been several reports on the tissue culture and micropropagation of *P. lactiflora* (Albers and Kunneman 1992; Gabryszewska 2010; Hosoki et al. 1989; Jana and Jeong 2013; Rather et al. 2014; Tian et al. 2010; Wu et al. 2011; Yu et al. 2012a, 2012b; Yu et al. 2013). Recently, it has been reported that sodium chloride pretreatment and adding polyvinyl pyrrolidone to the culture medium is effective against browning which is often a problem in peony tissue culture (Cai et al. 2020). Furthermore, propagation and transformation methods via callusing have also been studied in recent years (Duan et al. 2022; Lee et al. 2022; Song et al. 2023). Several reports among the above have described acclimation methods for cultured peony plantlets (Albers and Kunneman 1992; Hosoki et al. 1989; Rather et al. 2014). However, the survival rate of plantlets in these cases was about half which is not sufficient. A bio-nursery system using plant tissue culture techniques have been developing to solve the problem of peony seedling supply (Yoshimatsu et al. 2018) using PLKD2 clone derived from a seed of the Japanese medicinal cultivar ‘Kitasaisho’ (Hatakeyama et al. 1998). However, further improvements are needed to contribute to the commercial-scale seedling supply. In particular, the acclimation process of cultured plantlets is critical in seedling production using plant tissue culture techniques; however, there have been few reports on the acclimation method of cultured peony plantlets. It is also necessary to improve tissue culture conditions because some cultured strains are difficult to grow using previously reported methods. Therefore, in the present study, tissue culture conditions and methods of acclimation of *P. lactiflora* were investigated including recalcitrant clones/strains for tissue culture.

To establish a bio-nursery system for *P. lactiflora*, cultured shoots from several peony seeds and rhizomes were induced (Supplementary Table S1). Using these cultured shoots, we investigated the effects of temperature conditions on shoot proliferation and growth. Temperatures of 25°C have been used in several previous tissue culture studies on peony shoot proliferation (Gabryszewska 2010; Jana and Jeong 2013; Wu et al. 2011; Yu et al. 2012a, 2012b). On the other hand, it is also reported that 15°C was more suitable for shoot proliferation than 20°C in tissue culture for peonies (Albers and Kunneman 1992; Yoshimatsu et al. 2018). Initially, shoot propagation was done at 15°C in our studies; however, some of the culture clones grew poorly when the subculture was repeated at 15°C, making it difficult to maintain shoot cultures. Thus, the suitable temperature condition for shoot proliferation and peony growth was investigated by comparing two temperature conditions; constant 15°C or changing 15/5°C diurnal temperature. The results demonstrated that most of the clones had a higher number of shoots and better growth when incubated at 15/5°C (Figure 1). Among clones that detect a significant difference in the number of shoots, only PLKD2 also showed a statistically significant difference in shoot height (Supplementary Figure S1). As a result of transferring clones that were difficult to proliferate at 15°C to plant growth chamber of 15/5°C, the growths of these clones were restored and it became possible to maintain these cultures (Supplementary Figure S2). Under 15/5°C conditions, the length of the light period and light intensity differed from those under 15°C conditions. These factors may have influenced our results. However, because the temperature conditions of 15/5°C are similar to those at the sprouting time of peonies in the field, the effect of temperature is considered to be substantial.

**Figure 1:**
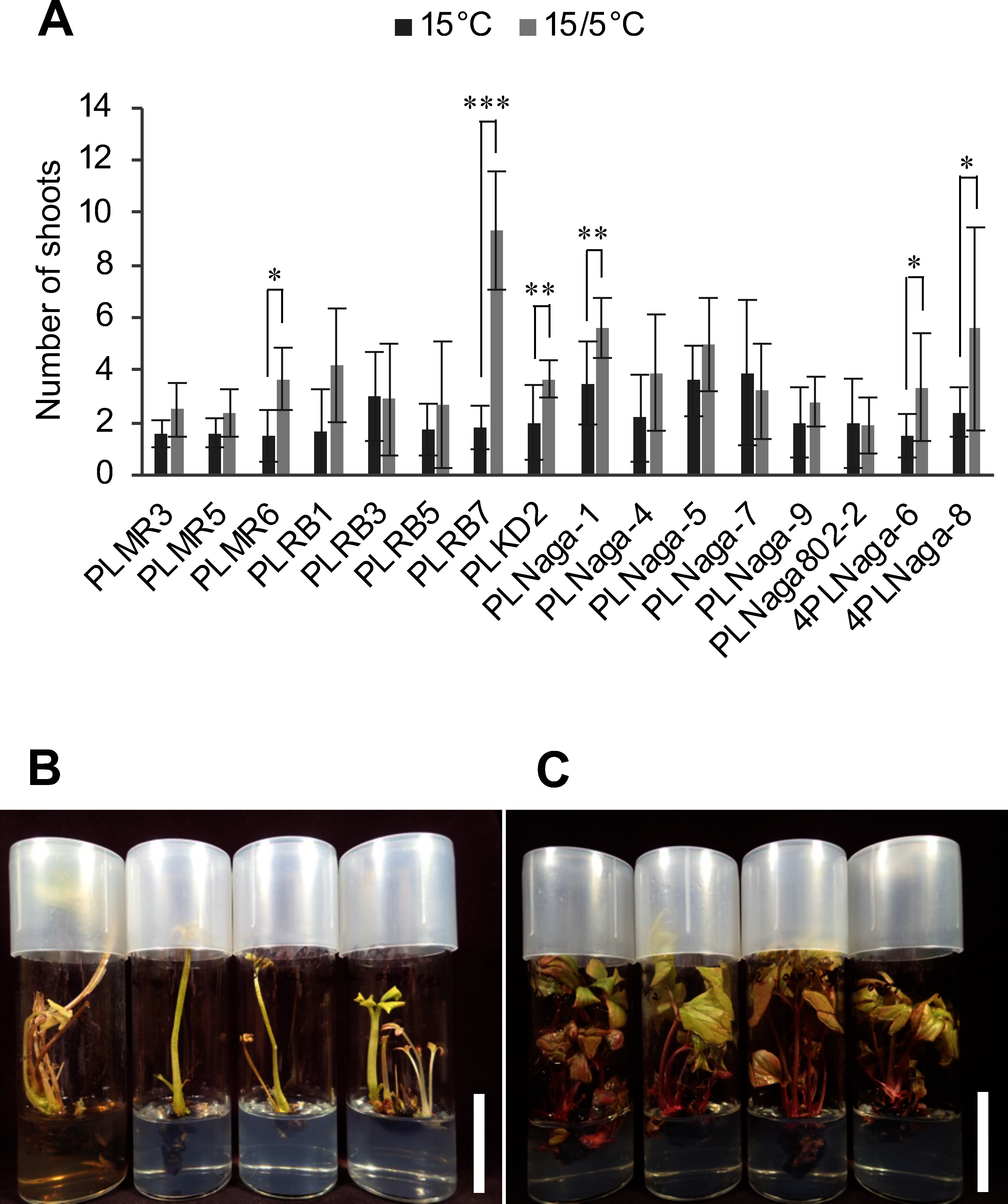
Figure 1. Comparison of shoot growth under different temperature conditions. Cultured peony shoots incubated for about three to four months were divided vertically. Thus prepared shoot segments were transferred to a half-strength macro element MS (1/2MS) medium containing 2% (w/v) sucrose, 6 mM Ca^2+^, 3 mg l^−1^ 6-benzyladenine, and 0.125% (w/v) gellan gum [(2)/2MS4CB3 medium] and incubated at a constant temperature of 15°C [14 h light period (fluorescent light, 40–70 µmol m^−2^ s^−1^)] or a changing diurnal temperature of 15/5°C [15°C, 12 h light (fluorescent light, 80–130 µmol m^−2^ s^−1^)/5°C, 12 h dark]. These procedures were repeated using inoculum shoot segments under the same temperature conditions to confirm reproducibility. The number of shoots with a shoot height of 1 cm or more (A) was compared after 85–92 days of culture. Growth comparisons were performed for second-generation transfers. The values represent the mean±SD of at least four replicates. Asterisks indicate statistically significant differences (Student’s *t*-test; * *p*<0.05, ** *p*<0.01, *** *p*<0.001). The images show PLKD2 shoots cultured for 86 days at 15°C (B) or 15/5°C (C). The scale bars indicate 3 cm.

Low temperature treatment before incubation has also been reported to promote shoot elongation (Albers and Kunneman 1992). In the above experiment, the number of shoots with a shoot height of 1 cm or more (large shoot) was compared. These shoots were considered to elongate after breaking dormancy. On the other hand, the number of shoots with a shoot height of less than 1 cm (small shoot) at 15°C was higher or similar at 15/5°C (Supplementary Figure S3). This result suggests that the low temperature of 5°C promoted bud dormancy breaks and shoot elongation, resulting in the increased large shoots, rather than simply an increase in shoot number under 15/5°C conditions. It is known that endogenous gibberellin content increases with cold treatment in several plants (Derkx et al. 1994; Song et al. 2019; Yamauchi et al. 2004; Zanewich and Rood 1995). Although not examined in the present study, gibberellins have been used for shoot induction and proliferation of peony tissue culture in several reports (Gabryszewska 2010; Hosoki et al. 1989; Jana and Jeong 2013; Rather et al. 2014; Tian et al. 2010; Wu et al. 2011; Yu et al. 2012a, 2012b). Since gibberellins have dormancy-breaking and shoot elongation effects (Hedden and Sponsel 2015; Liu and Sherif 2019), the gibberellin content of cultured peony seedlings may be increased under 15/5°C conditions.

It has been reported that increasing the Ca^2+^ concentration in a half-strength macro element Murashige and Skoog (1/2MS) medium is suitable for the growth of cultured peonies (Wu et al. 2011; Yoshimatsu et al. 2018). Moreover, 1/2MS medium with double the Ca^2+^ concentration (3 mM) has been used as a basic condition in several studies (Jana and Jeong 2013; Yu et al. 2012a, 2012b). It has been reported that 6 mM Ca^2+^ in the medium enhances root growth and makes leaves more robust when compared to 1.5 mM Ca^2+^ (Yoshimatsu et al. 2018). However, its effect on growth after transplantation into the soil is unknown. Therefore, in the present study, we examined the effects of different Ca^2+^ concentrations during root induction on growth after transplantation.

Rooted plantlets were obtained from peony shoots (PLKD2) cultured on root induction medium containing double-(3 mM) or quadruple-strength (6 mM) Ca^2+^ in 1/2MS medium, and the growth of the cultured plantlets was compared after transplanting to the soil. Although not statistically significant, the survival rate of plantlets cultured on 6 mM Ca^2+^ was higher than that of plantlets cultured on 3 mM Ca^2+^ in a closed greenhouse 76 days after transplanting to soil (Table 1). Furthermore, after cultivation of the acclimated peony plantlets in a closed greenhouse for approximately six months, they were cold-treated at 4°C for 198 days and the growth of the plants was compared. The results showed that plants rooted on 6 mM Ca^2+^ had a significantly higher fresh weight and number of buds (Table 2).

**Table 1:** Table 1. Effect of calcium concentration during root induction on the growth of acclimated plantlets after transplantation.

Calcium concentration (mM)	Survival rate (%)	Shoot length (cm)	Number of fresh leaves
3	38.9	13.8±2.4	3.0±1.1
6	76.9	15.1±2.6	2.8±0.9
*p*-value	0.067^a^	0.33^b^	0.69^b^

Shoot segments prepared as mentioned in Figure 1, transferred to 1/2MS media containing 2% (w/v) sucrose, 3 mM or 6 mM Ca^2+^, 0.5 mg l^−1^ indole-3-butyric acid, 0.125% (w/v) gellan gum [(2)/2MS2CIB0.5 or (2)/2MS4CIB0.5, respectively], and incubated at 15°C under 14 h light period (Pale Pink LED, 110–160 µmol m^−2^ s^−1^). Rooted plantlets (PLKD2) were cold-treated at 4°C for 72 days, then transplanted to a closed greenhouse [20°C, 60% relative humidity, 16 h light period (sunlight and supplemental LED light)]. Survival rate, shoot length, and number of fresh leaves were observed 76 days after transplantation. The values represent the mean±SD of at least seven replicates. ^a^ Fisher’s exact test, ^b^ Student’s *t*-test.

**Table 2:** Table 2. Effect of calcium concentration during root induction on the growth of acclimated peony plants after cold treatment.

Calcium concentration (mM)	Fresh weight (g)	Number of buds	Root length (cm)	Number of primary roots	Maximum root diameter (mm)
3	3.3±1.1	2.0±0.8	30.7±6.8	6.0±4.5	4.0±1.3
6	5.9±2.8	3.1±1.1	28.8±4.4	8.9±4.2	5.5±2.1
*p*-value^a^	0.019	0.041	0.506	0.189	0.127

After the peony plantlets (PLKD2: Ca^2+^ 3 mM or 6 mM) were cultivated in a closed greenhouse (20°C with the same conditions as Table 1) for 181 days, the aerial parts of the plants were removed and cold treated at 4°C for 198 days. After cold treatment, plant fresh weight, number of buds, root length, number of primary roots, and maximum root diameter were measured. The values represent the mean±SD of at least seven replicates. ^a^ Student’s *t*-test.

Ca^2+^ is one of the essential plant nutrients, which stabilizes cell walls and membranes and acts as a second messenger for various intracellular signaling (Thor 2019). Ca^2+^ application has also been reported to improve drought stress tolerance and increase root biomass (Hosseini et al. 2019; Naeem et al. 2018). In the present study, it may be that the increased Ca^2+^ concentration in the root induction medium improved the growth of cultured plantlets by strengthening cell walls and increasing drought stress tolerance. In Table 2, although no significant differences were observed, the number of primary roots and root diameter values were higher in 6 mM Ca^2+^, suggesting that Ca^2+^ promoted root growth, resulting in increased seedling weight.

Previously, a method for acclimating cultured peony plantlets to a plant growth chamber at 15°C has been reported (Yoshimatsu et al. 2018). However, because the number of seedlings that could be acclimated in the plant growth chamber was limited, it was necessary to scale-up the bio-nursery system to promote social implementation. Thus, a method of acclimation at 20°C was investigated to enable growth in a closed greenhouse, where more plantlets can be grown. A comparison of plantlet growth two months after transplantation showed that plantlets acclimated in a closed greenhouse at 20°C after approximately a month of cold treatment (AC2) grew better than those acclimated in the plant growth chamber at 15°C (AC1) (Figure 2). These results demonstrate that the acclimation of cultured peony plantlets is possible in a closed greenhouse, which enables the production of a larger number of peony seedlings than previous methods.

**Figure 2:**
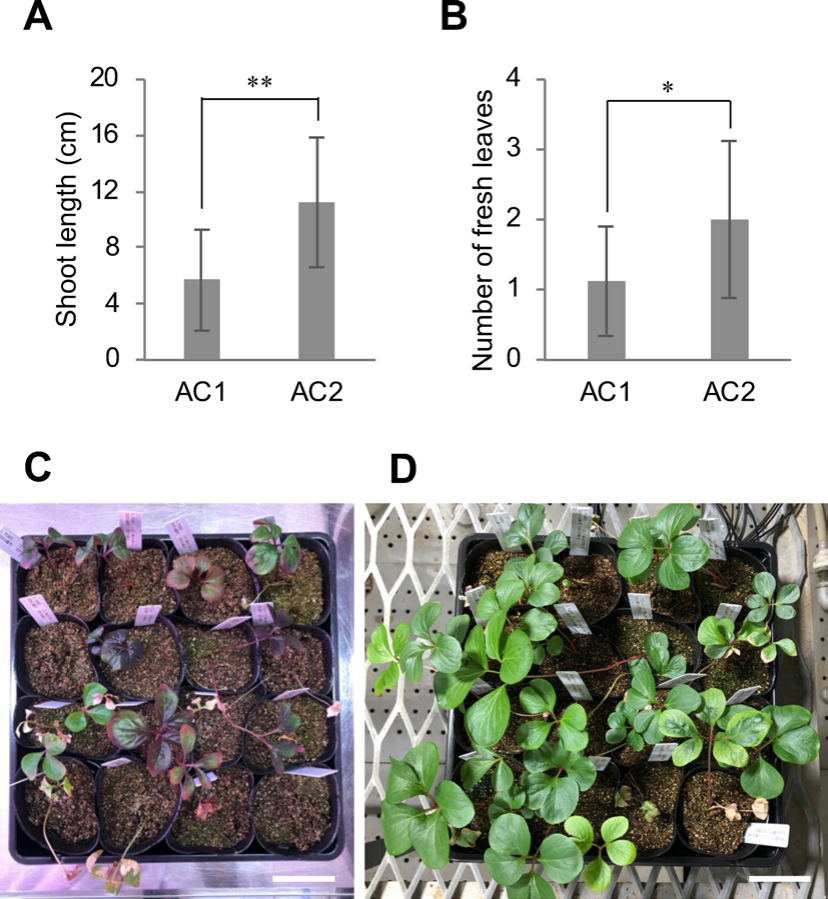
Figure 2. Examination of acclimation conditions for peony plantlets rooted in test tubes. Rooted plantlets (PLKD2, PLNa16-1 and PLNa16-10) acclimated by two different methods (AC1 and AC2) were transplanted into a plant growth chamber at 15°C, or closed greenhouse at 20°C after cold-treatment, and examined for shoot length (A) and number of fresh leaves (B) after two months from transplanting. The values represent the mean±SD (*n*=16). Asterisks indicate statistically significant differences (Student’s *t*-test; * *p*<0.05, ** *p*<0.01). Photographs show acclimated plants two months after transplantation (C: AC1, D: AC2). The scale bars indicate 5 cm. AC1: Acclimation in a plant growth chamber [15°C constant temperature, 70% relative humidity under 14 h light period (fluorescent light, 190–250 µmol m^−2^ s^−1^)]; AC2: Acclimation in a closed greenhouse (20°C with the same conditions as Table 1) after cold-treatment (4°C) of the rooted plantlets in the test tubes.

There were differences between AC1 and AC2 in temperature, light conditions, and cold treatment (Supplementary text). The plantlets acclimated under AC1 indicated poor shoot growth and reddish leaves (Figure 2C), suggesting they were stressed. Thus, it appeared that temperature conditions of 15°C are suitable for shoot propagation, but that 20°C is more suitable for subsequent shoot growth. In addition, the number of fresh leaves was higher in AC2 (Figure 2B). It is assumed that the cold treatment promoted bud dormancy breaks and shoot elongation, increasing the number of fresh leaves.

In this study, we found that the growth of peony cultured shoots improves under the 15/5°C diurnal temperature changing treatment, and that enhanced Ca^2+^ concentration during root induction has a significant effect on the growth after transplanting. Furthermore, it was found that healthy peony plantlets can be grown by transplanting into a closed greenhouse at 20°C after cold treatment. These findings will enable the propagation of peony strains that have been difficult to propagate via tissue culture and the more stable and efficient production of peony seedlings and are expected to contribute to the future supply of peony seedlings and expansion of domestic peony production. The molecular mechanisms of peony development and physiological responses are little understood. It is hoped that further studies will be conducted on the physiological aspects of these improved culture conditions and acclimation methods.

## Abbreviations

LED: light emitting diode
MS: Murashige and Skoog

## Description of supplementary files

Supplementary Table S1. List of cultured peony clones and their origins.

Supplementary Figure S1. Comparison of shoot height under different temperature conditions.

Supplementary Figure S2. The number of transferred shoot segments and shoot proliferation index at 15°C and 15/5°C.

Supplementary Figure S3. The number of small shoots under different temperature conditions.
