# Supplementary material for: Improvement of culture and acclimation conditions in a bio-nursery system for Paeonia lactiflora
Source: Plant Biotechnol (Tokyo). 2025 Jun 25;42(2):179–83. doi: 10.5511/plantbiotechnology.25.0120a (PMC12235421; doi:10.5511/plantbiotechnology.25.0120a)
Supplement: Supplementary Data [file plantbiotechnology-42-2-25.0120a-s001.pdf]

Supplementary Table S1. List of the cultured peony clones and their origins

Clone Name	Origin
**PLMR3**	Rhizome of *Paeonia lactiflora* ‘Martha Reed’ (horticultural cultivar)
**PLMR5**	Rhizome of *Paeonia lactiflora* ‘Martha Reed’ (horticultural cultivar)
**PLMR6**	Rhizome of *Paeonia lactiflora* ‘Martha Reed’ (horticultural cultivar)
**PLRB1**	Rhizome of *Paeonia lactiflora* ‘Rimbu’ (horticultural cultivar)
**PLRB3**	Rhizome of *Paeonia lactiflora* ‘Rimbu’ (horticultural cultivar)
**PLRB5**	Rhizome of *Paeonia lactiflora* ‘Rimbu’ (horticultural cultivar)
**PLRB7**	Rhizome of *Paeonia lactiflora* ‘Rimbu’ (horticultural cultivar)
**PLKD2**	Seed of *Paeonia lactiflora* ‘Kitasaisho’ (Japanese medicinal cultivar)
**PLNaga-1**	Seed of *Paeonia lactiflora* collected in Nagano prefecture in 2018
**PLNaga-4**	Seed of *Paeonia lactiflora* collected in Nagano prefecture in 2018
**PLNaga-5**	Seed of *Paeonia lactiflora* collected in Nagano prefecture in 2018
**PLNaga-7**	Seed of *Paeonia lactiflora* collected in Nagano prefecture in 2018
**PLNaga-9**	Seed of *Paeonia lactiflora* collected in Nagano prefecture in 2018
**PLNaga-13**	Seed of *Paeonia lactiflora* collected in Nagano prefecture in 2018
**PLNaga802-2**	Seed of *Paeonia lactiflora* collected in Nagano prefecture in 2018
**4PLNaga-4**	Seed of *Paeonia lactiflora* collected in Nagano prefecture in 2018
**4PLNaga-6**	Seed of *Paeonia lactiflora* collected in Nagano prefecture in 2018
**4PLNaga-8**	Seed of *Paeonia lactiflora* collected in Nagano prefecture in 2018
**PLNa16-1**	Rhizome of *Paeonia lactiflora* ‘Bonten’ (Japanese medicinal cultivar)
**PLNa16-10**	Rhizome of *Paeonia lactiflora* ‘Bonten’ (Japanese medicinal cultivar)

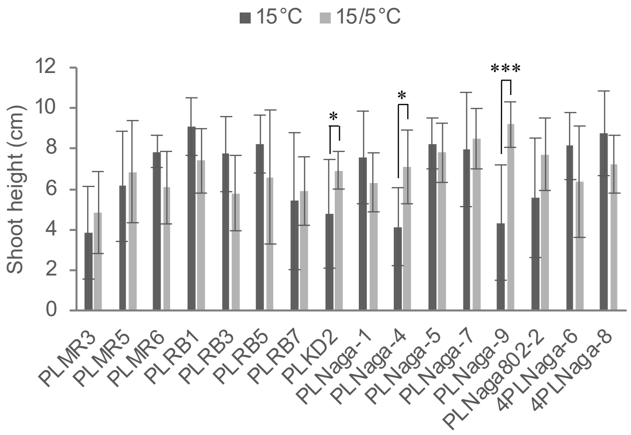

Supplementary Figure S1. Comparison of shoot height under different temperature conditions.

Shoot segments were transferred to (2)/2MS4CB3 media and incubated at 15°C or 15/5°C with the same conditions as Figure 1. Shoot height was compared after 85–92 days of culture. Growth comparisons were performed for second-generation transfers. The values represent the mean ± S.D. of at least four replicates. Asterisks indicate statistically significant differences (Student's *t*-test; * $p < 0.05$, ** $p < 0.01$, *** $p < 0.001$).

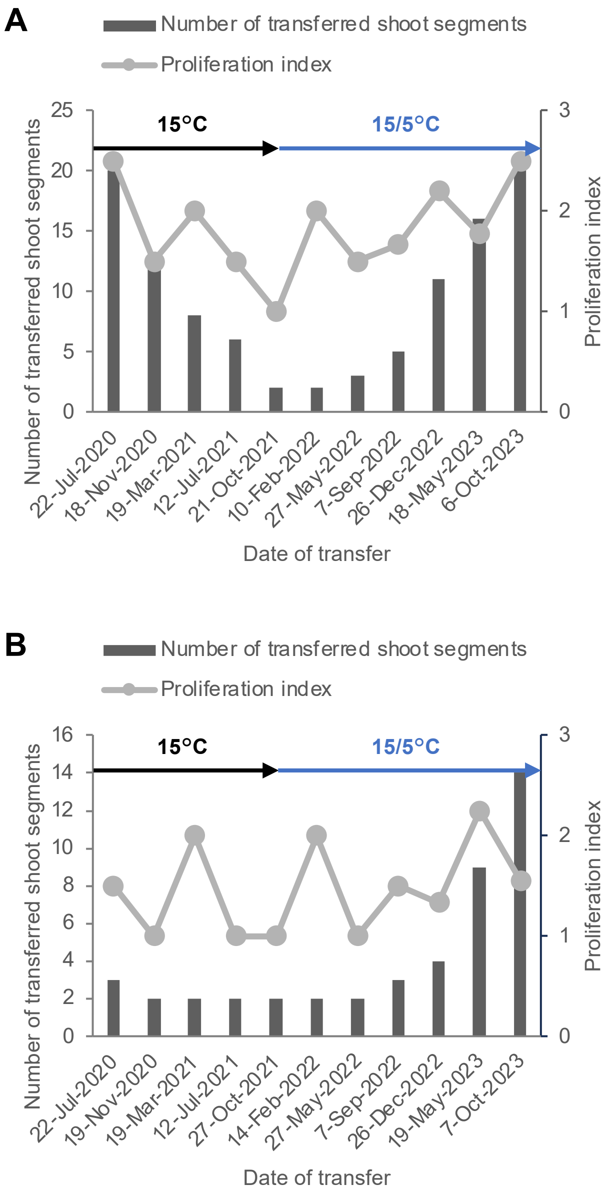

Supplementary Figure S2. The number of transferred shoot segments and shoot proliferation index at 15°C and 15/5°C

Shoot segments prepared from the cultured shoots (PLNaga-13 and 4PLNaga-4) were transferred to test tubes (one segment per tube) containing (2)/2MS4CB3 media and incubated at 15°C. After several transfers, culture conditions were changed to 15/5°C. Bars indicate the number of transferred shoot segments when the transfer is being conducted, and the line graph shows the proliferation index of PLNaga-13 (A) and 4PLNaga-4 (B). The proliferation index was calculated according to the following formula.

Proliferation index = Number of new test tubes transferred shoot segments / Number of test tubes contained material shoot cultures

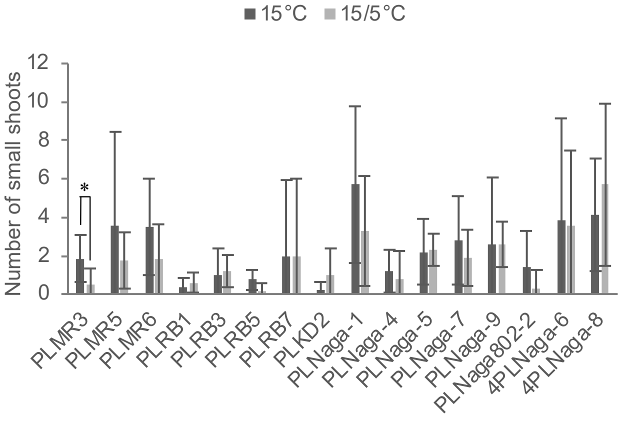

Supplementary Figure S3. The number of small shoots under different temperature conditions
Shoot segments were transferred to (2)/2MS4CB3 media and incubated at 15°C or 15/5°C with the same conditions as Figure 1. The number of shoots with a shoot height of less than 1 cm (small shoot) was compared after 85–92 days of culture. Growth comparisons were performed for second-generation transfers. The values represent the mean ± S.D. of at least four replicates. Asterisk indicates statistically significant difference (Student's *t*-test; * $p < 0.05$).

Supplementary text

Materials and methods

Plant material

A list of cultured peony clones and their origins used in this study is provided in Supplementary Table S1. PLKD2 was derived from a seed of the Japanese medicinal cultivar 'Kitasaisho' (Hatakeyama et al. 1998) grown in the field of Tsukuba Division, Research Center for Medicinal Plant Resources (RCMPR), National Institutes of Biomedical Innovation, Health and Nutrition (NIBN) (Yoshimatsu et al. 2018). The PLNaga series was derived from peony seeds collected at a farmer's field in Nagano, Japan, in 2018. PLMR and PLRB were derived from rhizomes of the horticultural cultivars 'Martha Reed' and 'Rimbu,' respectively, grown in the field of the Center for Medicinal Plant Resources, Toyama Prefectural Institute for Pharmaceutical Research. PLNa was derived from the rhizome of the Japanese medicinal cultivar 'Bonten,' grown in the Tsukuba Division, RCMPR, NIBN.

Induction of shoot cultures

The procedure for preparing a PLKD2 culture has been described previously (Yoshimatsu et al. 2018). Peony seeds were extracted by cracking and removing the seed coat. Seeds were then sterilized with 75% (v/v) ethanol for 1 min, rinsed with sterilized water, then sterilized in sodium hypochlorite with 3% effective chlorine concentration containing 0.1% (v/v) Tween-20 for 20 min. Seeds were then rinsed with sterilized water seven times, inoculated into 1/2MS medium (Duchefa Biochemie, M0233) containing 2% (w/v) sucrose, 3 mM Ca^{2+}, and 0.25% (w/v) gellan gum (Kelcogel®, SanEi Gen FFI) [(2)/2MS2C medium], then incubated at 20°C in the dark. After 40–50 days of incubation, only the embryo was transferred to 1/2MS medium containing 2% (w/v) sucrose, 6 mM Ca^{2+}, 3 mg l^{-1} 6-benzyladenine, and 0.125% (w/v) gellan gum [(2)/2MS4CB3 medium], then incubated at 15°C under 14 h light period [Pale Pink LED (Nippon Medical & Chemical Instruments Co., Ltd.), 110–160 μmol m^{-2} s^{-1}]. The Ca^{2+} concentration in the media was adjusted by adding a $CaCl_2$ solution.

Peony rhizomes were washed off with tap water and thoroughly rinsed with pure water, then sterilized with 100 times diluted kitchen bleach (Kao Corporation) for 20 min. They were buried in fine-grained vermiculite (Akagiengei Co., Ltd.), moistened with pure water, and stored at 4°C for one month. Explants containing buds were prepared from the rhizomes, sterilized with 75% (v/v) ethanol for 1 min, rinsed with sterilized water, and then sterilized in sodium hypochlorite with 3% effective chlorine concentration containing 0.1% (v/v) Tween-20 for 20 min. After rinsing the explants with sterilized water seven times, 2–6 mm height inocula containing shoot primordia were prepared and placed on (2)/2MS4CB3 media. These were incubated at 15°C under 14 h light period (fluorescent light, 40–70 μmol m^{-2} s^{-1}).

Shoot proliferation

Cultured peony shoots incubated for about three to four months were divided vertically. Prepared shoot segments (2–3 shoots per segment), which were transferred to (2)/2MS4CB3 media and incubated at 15°C under 14 h light period (fluorescent light, 40–70 µmol m^{-2} s^{-1}). Cultured shoots were transferred to fresh (2)/2MS4CB3 media every 3–4 months. After several transfers, the cultured shoots were used for subsequent experiments.

For the study of culture condition for shoot proliferation, shoot segments prepared as mentioned above were transferred to (2)/2MS4CB3 media and incubated at a constant temperature of 15°C [14 h light period (fluorescent light, 40–70 µmol m^{-2} s^{-1})] or a changing diurnal temperature of 15/5°C [15°C, 12 h light (fluorescent light, 80–130 µmol m^{-2} s^{-1}) / 5°C, 12 h dark]. The number of shoots with a shoot height of 1 cm or more and shoot height were observed after 85–92 days. These procedures were repeated using inoculum shoot segments under the same temperature conditions to confirm reproducibility.

Root induction

Cultured peony shoots incubated under a constant temperature condition were used for root induction. Shoot segments prepared as mentioned above, transferred to 1/2MS media containing 2% (w/v) sucrose, 3 mM or 6 mM Ca^{2+}, 0.5 mg l^{-1} indole-3-butyric acid, 0.125 % (w/v) gellan gum [(2)/2MS2CIB0.5 or (2)/2MS4CIB0.5, respectively], and incubated at 15°C under 14 h light period (Pale Pink LED, 110–160 µmol m^{-2} s^{-1}).

Acclimation of peony plantlets rooted in test tubes

For the examination of calcium concentration during root induction, rooted plantlets (PLKD2) in test tubes obtained with either (2)/2MS2CIB0.5 or (2)/2MS4CIB0.5 media were cold-treated at 4°C in the dark for 72 days and transplanted to polypots (7.5 cm diameter) filled with mixed soil [red ball soil, compost, nursing culture soil (Takii seed & Co., Ltd.), and river sand in a ratio of 6:2:1:1] and grown in a closed greenhouse [20°C, 60% relative humidity, 16 h light period (sunlight and supplemental LED light)]. The aerial parts of the plantlets were covered with a transparent plastic film to maintain high relative humidity. The film was gradually removed over approximately a week. An acclimation nutrient solution (3.0 mM $CaCl_2$, 1.5 mM $MgSO_4$) was applied for a week after planting and then changed to the nutrient solution into the Otsuka A formula (OAT Agrio Co., Ltd.) at 1/4 concentration. Survival rate, shoot length, and number of fresh leaves were observed at 76 days after transplanting. After peony plantlets (PLKD2) were cultivated in a closed greenhouse (20°C) for 181 days, the aerial parts of the plants were removed and cold-treated at 4°C for 198 days. After cold treatment, plant fresh weight, number of buds, root length, number of primary roots, and maximum root diameter were measured.

For the examination of acclimation method, plantlets (PLKD2, PLNa16-1, and PLNa16-10) rooted

on either (2)/2MS2CIB0.5 or (2)/2MS4CIB0.5 media were transplanted to polypots (7.5 cm diameter) filled with fine-grained vermiculite (Akagiengei Co., Ltd.) and grown in a plant growth chamber (LPH-411SPC, Nippon Medical & Chemical Instruments Co., Ltd.) [15°C constant temperature, 70% relative humidity under 14 h light period (fluorescent light, 190–250 µmol m^{-2} s^{-1})]. The aerial parts of the plantlets were covered with a transparent plastic lid to maintain high relative humidity and it was gradually removed over approximately a month. After transplanting, an acclimation nutrient solution was applied for one month and then changed to the Otsuka A formula at a 1/16 concentration (acclimation method 1: AC1). In acclimation method 2 (AC2), rooted plantlets in test tubes were cold-treated at 4°C in the dark for approximately a month and then transplanted to a closed greenhouse (20°C). The aerial parts of the plantlets were covered with a transparent plastic lid to maintain high relative humidity. The lid was gradually removed approximately a month. The growing soil and fertilizer conditions were the same as those for AC1.

References

Hatakeyama Y, Kumagai T, Katsuki S, Homma N, Ishizaki S, Miura T, Sawai K, Yamagishi T, Nishizawa M, Hayashi T (1998) Studies on Cultivation and Breeding of *Paeonia lactiflora* PALLAS (1) Characteristics of a New Medicinal Cultivar" Kitasaishou". *Nat Med* 52: 103–108 (in Japanese)

Yoshimatsu K, Kawano N, Inui T (2018) Peony strain, peony strain proliferation method, and peony cultivation method. Japan Patent 7060228B2, URL: https://www.j-platpat.inpit.go.jp/c1801/PU/JP-7060228/15/en (accessed on May 20, 2024)
